# Real-Time Topology-Aware Branch Segmentation for UAV Perception in Natural Environments

**DOI:** 10.3390/s26144628

**Published:** 2026-07-21

**Authors:** Tong Wang, Zhengran Zhou, Abner Asignacion, Satoshi Suzuki

**Affiliations:** Graduate School of Engineering, Chiba University, 1-33 Yayoi-cho, Inage-ku, Chiba 263-8522, Japan

**Keywords:** UAV perception, real-time segmentation, topological perception, tree branch analysis

## Abstract

**Highlights:**

**What are the main findings?**

- The proposed SSPPM, reparameterized GCBlock, and BOM effectively improve elongated branch representation, boundary quality, and deployment efficiency, enabling the segmentation network to achieve 89.94% mIoU on the Drone-Branch dataset while maintaining a real-time latency of 13.7 ms on the NVIDIA Jetson Orin Nano.
- The proposed topology-aware structural analysis effectively removes branch junctions and multi-branch regions, preserving structurally stable branch segments and improving the reliability of branch perception in complex natural environments.

**What is the implication of the main finding?**

- The proposed framework enables real-time branch segmentation, while topology-aware structural analysis improves the reliability of extracted branch structures, for stereo vision systems operating under limited onboard computational resources.
- By combining semantic segmentation with topology-aware structural analysis, the proposed method provides an effective solution for extracting stable branch structures, supporting safe UAV approach, obstacle avoidance, environmental inspection, and other downstream perception tasks.

**Abstract:**

Autonomous UAVs operating in forest environments require accurate branch perception to enable reliable environmental understanding for downstream tasks such as navigation, environmental inspection, and obstacle avoidance. However, existing semantic segmentation methods primarily focus on pixel-wise classification and often fail to preserve the structural topology of branches, making it difficult to distinguish reliable branch segments from complex regions such as junctions and overlapping structures. In addition, achieving robust real-time performance on resource-constrained onboard platforms remains challenging due to the slender and irregular characteristics of natural branches. To address these challenges, this paper proposes a topology-aware branch perception framework that integrates real-time efficient semantic segmentation with structural topology analysis. For branch segmentation, a Strip-Swift Pyramid Pooling Module (SSPPM) is introduced to enhance elongated structure representation through progressive pooling and strip-based directional context aggregation. A reparameterized Golden Cudgel Block (GCBlock) and a Boundary Optimization Module (BOM) are further incorporated to improve deployment efficiency and boundary quality. Building upon the predicted segmentation masks, a topology-aware structural analysis module is developed to identify and remove branch junctions and multi-branch regions through skeleton-based connectivity analysis, preserving only structurally stable branch segments for subsequent perception and analysis. This strategy improves the robustness and consistency of branch representation in cluttered natural environments. Experimental results demonstrate that the proposed method achieves 89.94% mIoU on the Drone-Branch dataset. When deployed on the NVIDIA Jetson Orin Nano with TensorRT acceleration, the proposed segmentation network achieves a stable inference latency of 13.7 ms. The topology-aware structural analysis is subsequently applied as a post-processing stage to improve the reliability of extracted branch structures. These results demonstrate the effectiveness of the proposed framework for branch perception on resource-constrained UAV platforms.

## 1. Introduction

### 1.1. Background

Autonomous UAVs operating in forest environments require reliable perception of tree branches to support safe navigation and obstacle avoidance [1,2,3,4,5]. When UAVs fly close to vegetation, slender branches and cluttered backgrounds significantly affect perception reliability and flight safety. Accurate branch perception is therefore a fundamental prerequisite for downstream flight control and decision-making.

Natural tree branches exhibit slender geometries, irregular topologies, frequent occlusions, and complex bifurcation structures [6,7,8]. These characteristics make robust branch perception challenging, especially under the limited computational resources of embedded UAV platforms. In particular, branch junctions and overlapping structures often produce ambiguous visual patterns that degrade segmentation quality and complicate subsequent structural analysis.

Consequently, the problem extends beyond generic branch segmentation to topology-aware branch representation. Reliable perception requires not only pixel-wise classification but also the ability to distinguish stable branch segments from structurally complex regions such as bifurcations and overlapping branches. Conventional object detectors based on bounding boxes cannot adequately capture these geometric properties. In contrast, semantic segmentation provides dense structural information and has demonstrated strong potential for thin-structure perception tasks such as road extraction, vessel segmentation, and branch analysis [9,10,11,12,13].

Despite recent advances in lightweight semantic segmentation, existing methods often fail to preserve the structural continuity of slender branches under embedded deployment constraints [14,15,16]. Conventional square receptive fields are not well suited for modeling the directional continuity and anisotropic geometry of branches, resulting in fragmented predictions and unstable structural representations. Moreover, few studies explicitly incorporate topology-aware reasoning into the segmentation pipeline to identify and remove structurally ambiguous regions, such as branch junctions and multi-branch areas [17,18].

Given the research gap in preserving branch topology and filtering structurally unstable regions from segmentation results, this paper focuses on the visual perception problem of topology-aware branch representation based on real-time semantic segmentation. The proposed method generates refined branch masks with unstable regions removed, providing more reliable structural inputs for downstream navigation and obstacle avoidance. Camera motion estimation, target tracking, motion planning, and physical interaction are beyond the scope of this study.

Hence, this study proposes a real-time topology-aware branch perception framework for onboard UAV systems. The main contributions of this study are as follows:

(1) Strip-Swift Pyramid Pooling Module (SSPPM):
  We propose an SSPPM for the effective multiscale representation of elongated branch structures. It replaces conventional parallel pyramid designs with a progressive pooling strategy while introducing a dedicated strip pooling mechanism for anisotropic context modeling. This design captures long-range directional continuity and improves computational efficiency by reducing GPU load imbalance, yielding consistent accuracy improvements across datasets.
(2) Deployment-Friendly Backbone with Edge Refinement: We develop a high-efficiency backbone that integrates reparameterized golden cudgel blocks (GCBlocks) with a boundary-optimization module (BOM). The architecture leverages multibranch training for enhanced representation while collapsing into a streamlined single-path structure during inference, thus achieving improved boundary precision for slender targets without additional deployment overhead.
(3) Topology-Guided Structural Filtering Framework: We propose a topology-guided filtering pipeline that bridges semantic segmentation and structural reasoning. Through skeleton-based topology analysis, the framework identifies and removes structurally unstable regions—such as bifurcations, junctions, and overlapping areas—from the segmentation mask, while preserving topologically reliable branch segments. This filtering strategy improves the structural quality and reliability of branch masks for embedded UAV perception.

The remainder of this paper is organized as follows. Section 1 reviews the background of UAV perception and real-time semantic segmentation for thin-structure perception. Section 2 presents the proposed framework, including the overall system architecture, the improved DDRNet for real-time branch segmentation with the proposed GCBlock, SSPPM, and BOM modules, as well as the topology-guided structural filtering strategy. Section 3 presents the experimental results, including implementation details, ablation studies, comparative evaluations, edge deployment performance, and qualitative and quantitative validation of the proposed topology-guided structural filtering framework. Finally, Section 4 concludes the paper and discusses future research directions.

### 1.2. Related Work

Recent advancements in computer vision have significantly expanded the capacity of autonomous systems to perceive and model intricate natural environments. Robust perception of tree branches in cluttered and unstructured scenes remains a fundamental challenge, particularly for UAV navigation and obstacle avoidance in forest environments [19,20]. In response to these challenges, recent research has begun to shift from generic object detection toward structural and topology-aware perception, emphasizing the reconstruction of geometrically consistent and topologically stable representations rather than isolated object instances.

#### 1.2.1. Branch Perception and Structural Analysis

Branch perception has been widely studied in agricultural and forestry robotics, primarily for applications such as pruning automation, yield estimation, and structural inspection [21,22]. For example, Albaroudi et al. [21] proposed a branch geometry estimation framework that reconstructs hierarchical tree structures for robotic pruning, enabling explicit modeling of branch connectivity. Similarly, Sun et al. [22] developed a real-time trunk and branch detection method for pear trees, focusing on robust localization of upright structures under natural orchard conditions. These methods demonstrate the feasibility of structured branch understanding in controlled agricultural environments.

Early work in this domain relied on handcrafted features and classical vision pipelines, while recent approaches have shifted toward deep learning-based semantic and instance segmentation. For instance, Kefalas et al. [23] proposed a vision-based pruning system that performs fine-grained branch segmentation and estimates precise pruning points, improving automation accuracy in structured tree environments. Dukić et al. [24] further integrated RGB-D reconstruction with automated annotation strategies to support supervised learning of branch structures, reducing manual labeling effort and improving dataset scalability. Tong et al. [7] designed an end-to-end pruning robot system that combines branch segmentation with decision-making control for apple tree pruning tasks.

Although these methods improve visual recognition accuracy, they generally treat branches as independent pixel regions without explicitly enforcing structural continuity. To address this limitation, related studies in thin-structure perception have emphasized the importance of preserving topology in elongated and high-aspect-ratio objects. For example, Li et al. [25] proposed Branch-YOLO for efficient detection of thin structures, improving sensitivity to low-width objects. In UAV navigation scenarios, Fathi et al. [26] introduced a depth-aware segmentation framework that enhances obstacle continuity reasoning in cluttered environments. Tao et al. [27] proposed a hybrid Transformer–CNN segmentation network with connectivity structures for road extraction, enabling more complete reconstruction of elongated road networks. Chen et al. [28] developed an edge-aware crack segmentation network that integrates attention mechanisms to preserve boundary continuity and improve the perception of fine-scale crack structures. Wei et al. [29] presented a multi-level attention framework in the Hough domain for power line segmentation, enhancing the robustness of long-range linear structure perception in complex scenes. Similarly, Bilal et al. [30] demonstrated that topology-preserving architectures significantly improve vessel continuity in medical image segmentation, highlighting the general importance of structural consistency in thin-object perception.

Despite these advances, most existing methods still lack explicit modeling of global geometric and topological constraints. Conventional bounding-box detectors fail to capture connectivity, while standard segmentation approaches often neglect global structural consistency. As a result, reliable perception of natural branching systems remains challenging, particularly in scenarios involving occlusions, bifurcations, and irregular branching patterns.

#### 1.2.2. Real-Time Thin-Structure Segmentation

Reliable branch perception requires real-time semantic segmentation that can operate on resource-constrained embedded UAV platforms. Existing real-time segmentation architectures generally reflect two major paradigms.

(1) Single-branch Architectures:
  Models such as STDC [31] and Fast-SCNN [32] prioritize inference efficiency through lightweight backbones and aggressive downsampling strategies. Although these architectures achieve high frame rates, they typically sacrifice spatial continuity in deeper layers, thus resulting in fragmented predictions for slender branch structures.
(2) Multi-branch Architectures: Methods such as BiSeNet [33] and DDRNet [34] employ bilateral pathways to simultaneously preserve spatial details and semantic context. While these architectures improve boundary quality, their frequent feature-fusion operations and complex memory-access patterns may introduce additional computational overhead during deployment.

Despite their architectural differences, most existing segmentation frameworks rely predominantly on isotropic square receptive fields (e.g., 3×3 and 7×7 kernels). However, such receptive fields are not suitable for the highly directional and anisotropic geometry of natural branches [35]. Consequently, existing lightweight models fail to preserve the topology continuity and directional consistency of elongated branch structures under real-time deployment constraints.

Furthermore, most current branch-perception pipelines focus primarily on semantic recognition while largely overlooking the integration of topology-aware reasoning to refine segmentation outputs, leaving fragmented predictions and ambiguous regions—such as branch junctions and overlapping areas—unresolved in the final results.

Research Gap

In summary, although significant progress has been achieved in branch perception and real-time semantic segmentation, several critical limitations remain.

(1) Inadequate multiscale representation for anisotropic branch structures. Existing lightweight segmentation models rely on isotropic square receptive fields, thus resulting in fragmented predictions for slender branches and failing to capture long-range directional continuity.
(2) Boundary precision vs. deployment efficiency in dual-path architectures. Current dual-path models improve context but necessitate frequent fusion operations and incur memory overheads, thus rendering them inefficient in terms of boundary refinement for slender targets on embedded platforms.
(3) Limited integration of topology-aware reasoning into segmentation pipelines. Existing methods mainly terminate at semantic segmentation and do not exploit branch topology to refine their outputs by identifying and removing structurally unstable regions—such as bifurcations and overlapping areas—from segmentation results.

These limitations warrant a unified framework that jointly enables anisotropic multiscale perception, efficient boundary-aware segmentation, and topology-guided structural refinement for resource-constrained UAV perception.

## 2. Materials and Methods

### 2.1. System Architecture and Workflow

**Figure 1:**
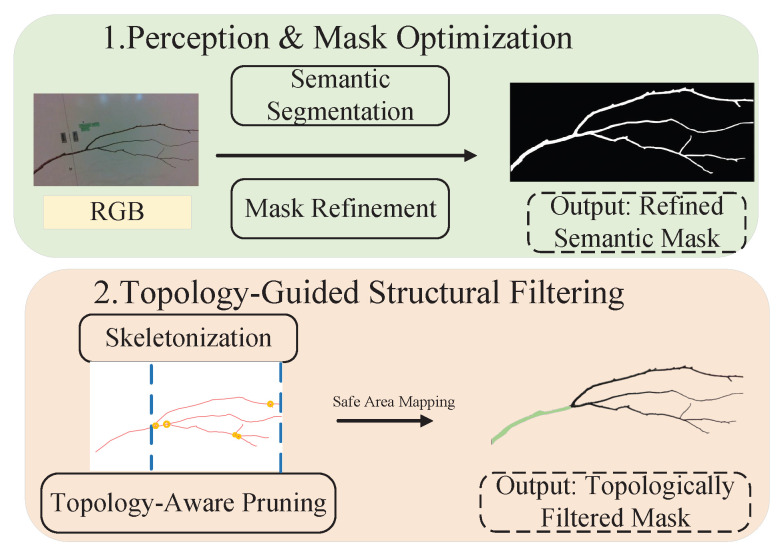
Structure of the proposed system. The blue dashed region indicates the area where multiple branches overlap, the yellow circles denote detected branch junctions, and the green shaded region represents the final retained safe region after topology-guided structural filtering.

The proposed system establishes a robust pipeline for real-time branch perception in complex natural environments. As illustrated in Figure 1, the framework integrates deep-learning-based segmentation with topology-guided structural refinement to generate reliable branch masks from RGB input. The system comprises two modular stages: (1) perception and mask optimization, and (2) topology-guided structural filtering.

Figure 1 illustrates the workflow of the proposed framework. The perception module extracts branch regions from the RGB input and performs mask refinement. Subsequently, the refined mask is skeletonized for topology analysis, which identifies and removes structurally unstable regions—such as bifurcations, junctions, and overlapping areas—while preserving topologically reliable branch segments. The final output is a refined branch mask with unstable regions excluded, providing more reliable structural input for downstream navigation and obstacle avoidance.

(1) Perception and Mask Optimization: To accommodate the slender and multiscale nature of tree branches, the system utilizes a lightweight backbone enhanced by the proposed Strip-Swift Pyramid Pooling Module (SSPPM). This stage yields a high-fidelity semantic mask that captures long-range directional context, while a Boundary Optimization Module (BOM) ensures sharp edge definition, providing precise inputs for subsequent structural filtering.
(2) Topology-Guided Structural Filtering: Because branch junctions and overlapping structures often produce ambiguous visual patterns that degrade segmentation quality, this module evaluates the skeletonized mask to identify structurally unstable components. By analyzing local connectivity and transition density, the system detects topological singularities—such as bifurcation points, junctions, and densely overlapping regions—and removes the corresponding areas from the original mask. Only skeleton segments that exhibit reliable structural characteristics are retained as indicators of stable branch regions, while the associated mask regions are preserved. The remaining unstable regions are filtered out, yielding a refined branch mask with improved structural reliability.

The proposed framework terminates at the generation of a refined branch mask, in which structurally ambiguous regions have been removed. This output can serve as a reliable environmental prior for downstream applications such as UAV navigation and obstacle avoidance. Camera motion estimation, target tracking, motion planning, and physical interaction are beyond the scope of this study.

### 2.2. Proposed Branch Segmentation Method

**Figure 2:**
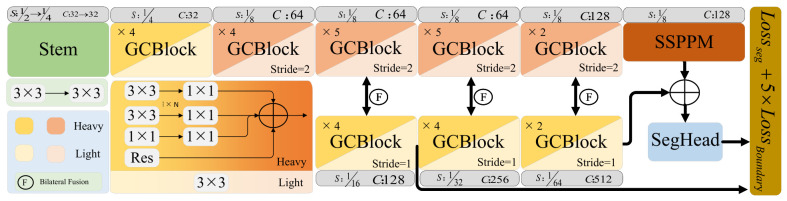
The structure of the improved DDRNetBrnach. The dark-colored blocks represent the training-time architecture (Heavy), while the light-colored blocks represent the inference-time architecture (Light) after structural re-parameterization.

As illustrated in Figure 2, the proposed model retains the core dual-path skeleton and bilateral fusion mechanism of DDRNet. To satisfy the stringent real-time requirements of drone-borne platforms, we introduce three strategic enhancements:

(1) GCBlock for efficient feature extraction,
(2) SSPPM for balanced multi-scale context aggregation, and
(3) BOM with focal loss to refine the structural contours.

#### 2.2.1. GCBlock

To enhance feature-extraction capabilities while maintaining real-time inference speed, the GCBlock [36] is integrated into the DDRNetBranch. The core philosophy of GCBlock is structural reparameterization, which is characterized by the principle of “self-enlargement during training and self-contraction during inference.”

As illustrated in Figure 2, the GCBlock adopts a complex multiconvolution and multipath architecture during the training phase. By employing vertical convolution stacking (e.g., 3×3 convolution followed by 1×1 convolution) and horizontal multibranch parallelism, the block significantly strengthens the network’s representational ability. This design enables the model to function as an “internal teacher,” thereby effectively capturing deep semantic features and precise boundary information without requiring external pretrained models or complex distillation schemes.

Despite its complex training structure, the GCBlock is simplified through mathematically equivalent transformations prior to inference. First, the convolution layers are fused with their subsequent BatchNorm layers. Subsequently, through vertical fusion (combining sequential 3×3 and 1×1 kernels) and horizontal summation (adding weights from all parallel paths), the entire module collapses into a single, standard 3×3 convolutional layer. This process eliminates the memory-access cost typically associated with multipath structures, thereby ensuring superior inference efficiency.

Within the DDRNetBranch architecture, the GCBlock is deployed with different strides to satisfy specific branch requirements: Semantic Branch: The GCBlock operates with a stride of 2. In this configuration, it performs efficient downsampling while expanding the receptive field to capture the global semantic context. Detailed Branch: The GCBlock operates with a stride of 1. This branch maintains high-resolution feature maps, with emphasis on the extraction of fine-grained boundaries and geometric details through the diverse paths of the GCBlock.

#### 2.2.2. SSPPM

The original deep aggregation pyramid pooling module (DAPPM) captures multiscale contextual information by combining multiple parallel branches with heterogeneous kernel sizes, strides, and upsampling operations. Each branch extracts features at a specific receptive field, and the resulting outputs are upsampled and added element-wise for fusion. Although effective in enriching representation, this fragmented multibranch design presents several disadvantages. Specifically, the heterogeneous computational demand across branches result in GPU load imbalance; some computing units are underutilized, whereas others are overloaded. Moreover, the repeated use of large-kernel convolutions and upsampling introduces redundant operations and a significant memory-bandwidth overhead, which consequently degrades the inference efficiency.

**Figure 3:**
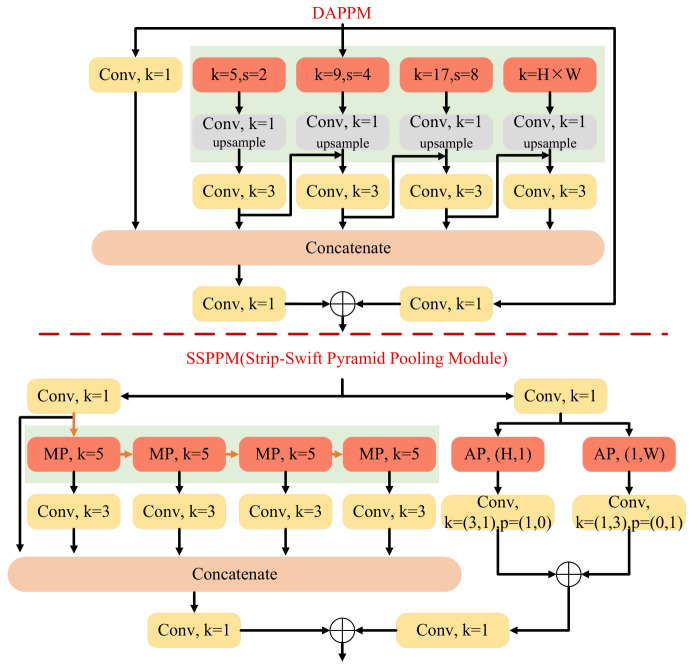
Strip-Swift Pyramid Pooling Module (SSPPM).

To overcome these limitations and further enhance the capture of long-range dependencies, we propose a SSPPM, as shown in Figure 3. The SSPPM reformulates the DAPPM by replacing the fragmented parallel branches with a streamlined serial pooling–convolution pipeline while simultaneously introducing a dedicated strip-pooling branch to manage complex, anisotropic spatial contexts.

To address the GPU load imbalance, the SSPPM first utilizes a “swift” serial pipeline. It employs four successive 5×5 max-pooling layers (stride = 1, padding = 2) to ensure that spatial resolution is preserved while the receptive fields are gradually expanded. The equivalent receptive field of the *k*-th layer can be computed using the following recursive formulation:

(1) $$l_{k}=l_{k-1}+\left(\left(f_{k}-1\right)*\prod_{i=1}^{k-1}s_{i}\right)$$

where $l_{k}$ denotes the receptive field at the *k*-th layer, $l_{k-1}$ the receptive field of the previous layer, $f_{k}$ the kernel size of the *k*-th operation, and $s_{i}$ the stride of layer *i*. Because pooling was is conducted with a stride $s_{i}=1$, the effective receptive field expands linearly. For example, stacking *n* layers of 5×5 pooling yields 5, 9, 13, and 17 receptive fields for n=1,2,3,4, respectively. Following each pooling operation, a 3×3 convolution is applied to densify feature extraction. This avoids the sparse computation characteristics of large-kernel convolutions and strided operations in the original DAPPM, thus achieving equivalent multiscale coverage at a substantially lower cost.

Although the serial pooling pipeline efficiently captures local and regional contexts, it relies on square windows. Using large, square pooling windows cannot accommodate objects that have long-range banded or discretely distributed structures because they inevitably incorporate contaminated information from irrelevant regions. Hence, a strip pooling branch is integrated into the SSPPM. Instead of a square kernel, strip pooling requires the spatial pooling extent of (H,1) or (1,W). By averaging all feature values in a row or column, it deploys a long kernel shape along one spatial dimension to capture long-range relations while maintaining a narrow shape along the other to prevent irrelevant regions from interfering. The input undergoes average pooling along both horizontal and vertical dimensions, and the results are input to one-dimensional convolutional layers with a kernel size of 3 to modulate the current location and its neighboring features.

Prior to processing, a 1×1 convolution reduces the channel dimensionality, thereby alleviating the computational overhead. Finally, the outputs from the initial 1×1 branch, the multiscale serial cascade, and the horizontal/vertical strip pooling layers are concatenated together. The final 1×1 convolution combines these diverse spatial and semantic embeddings into a compact representation.

In summary, the SSPPM maintains its ability to capture multiscale contextual cues while significantly improving efficiency and representational ability. By combining a serial pooling–convolution pipeline with anisotropic strip pooling, it achieves balanced GPU utilization; avoids the noise of large, square pooling windows; and offers faster, more accurate inferences.

#### 2.2.3. BOM

Accurately defining the boundaries between slender and irregular branches for branch removal or manipulation is a significant challenge in autonomous drone operations. Owing to the complex backgrounds and inherent thinness of branches, standard segmentation losses typically result in blurred contours and thus imprecise candidate box localization. Hence, we propose a BOM. This design is inspired by two key studies: the joint segmentation and boundary-detection framework proposed in [37] and the focal-loss formulation introduced in [38].

The first study [37] presents a dual-branch network with a shared backbone that enables semantic segmentation and boundary detection to interact across multiple scales. This joint learning paradigm allows boundary information to substantially enhance the structural accuracy and contour sharpness of segmentation results, while semantic cues from segmentation simultaneously improve the accuracy of boundary detection. The second study [38] reformulates the standard cross-entropy loss by introducing a modulating factor that dynamically downweights easy-to-classify samples (e.g., abundant background pixels), thus causing the training to prioritize difficult or rare samples. This approach effectively addresses the class-imbalance problem and improves the model’s ability to capture rare and informative features.

Based on these insights, we integrate focal loss into the boundary-detection branch of our framework. In branch perception tasks, boundary pixels are much sparser than nonboundary pixels, and conventional cross-entropy loss tends to bias the model toward background regions, thus resulting in blurred or inaccurate contours for slender branches. By contrast, focal loss directly mitigates this imbalance by prioritizing harder, less-represented boundary samples.

To precisely evaluate edge confidence, the predicted edge probability map $P=\{p_{i}\}$ is derived from the normalized prediction uncertainty (pixel-wise entropy) of the segmentation logits. Ground-truth edge masks $Y=\left\{y_{i}\right\}\in \left\{0,1\right\}$ are automatically generated from segmentation label maps. Specifically, an 8-directional spatial difference operator is executed to identify label transitions across neighboring pixels, producing a 1-pixel-wide initial boundary mask. To account for annotation tolerance, this mask is dilated using a 2D max-pooling operator with a kernel size of $k_{\mathrm{edge}}=5$ pixels. To prevent boundary artifacts during spatial filtering, a border margin of $k_{\mathrm{edge}}+1=6$ pixels along the image boundaries is set to zero.

Rather than computing supervision exclusively on boundary pixels, the binary focal loss $L_{\mathrm{boundary}}$ is calculated across all valid pixels in the image to explicitly penalize false positives in background regions. The full binary focal loss formulation is expressed as:

(2) $$L_{\mathrm{boundary}}=-\frac{1}{N_{\mathrm{valid}}}\sum_{i\in \Omega_{\mathrm{valid}}}\alpha_{t,i}{\left(1-p_{t,i}\right)}^{\gamma }\log\left(p_{t,i}\right)$$

where $\Omega_{\mathrm{valid}}$ represents the set of non-ignored valid pixels, $N_{\mathrm{valid}}=\left|\Omega_{\mathrm{valid}}\right|$, and $y_{i}\in \left\{0,1\right\}$ is the ground-truth boundary label. The term $p_{t,i}$ represents the target-aligned predicted probability:

(3) $$p_{t,i}=\left\{\begin{array}{cc} p_{i}, & \mathrm{if}\;y_{i}=1\\ 1-p_{i}, & \mathrm{if}\;y_{i}=0 \end{array}\right.$$

and $\alpha_{t,i}$ is the class-balancing weight defined as:

(4) $$\alpha_{t,i}=\left\{\begin{array}{cc} \alpha , & \mathrm{if}\;y_{i}=1\\ 1-\alpha , & \mathrm{if}\;y_{i}=0 \end{array}\right.$$

where α=0.25 balances the sparse boundary pixels ($y_{i}=1$) against the abundant non-boundary background pixels ($y_{i}=0$), and γ=2.0 suppresses easy background samples. This binary formulation allows the model to effectively suppress background false positives while maintaining sharp boundary definitions.

The final objective function is expressed as:

(5) $$L_{\mathrm{total}\;}=L_{\mathrm{seg}}+\lambda_{\mathrm{edge}}\cdot L_{\mathrm{boundary}\;}$$

where $L_{\mathrm{seg}}$ is the segmentation loss, and the boundary loss weight is set to $\lambda_{\mathrm{edge}}=5.0$ to ensure adequate supervision for edge refinement.

**Figure 4:**
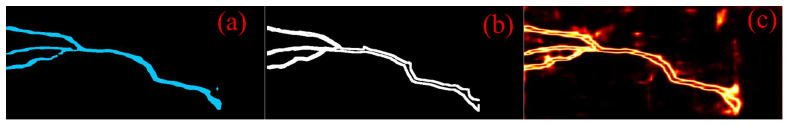
Visualization of boundary optimization module (BOM). (**a**) Final predicted segmentation result; (**b**) ground-truth edge map ($GT_{edge}$) used for supervision; (**c**) predicted edge confidence map (entropy).

To illustrate the role of BOM more effectively, Figure 4 presents the intermediate edge-detection results derived from the context branch. This branch leverages deep semantic features to achieve a robust category representation. Subfigure (a) presents the final segmentation output, where the contours of slender branches are expected to be sharp and continuous. Subfigure (b) shows the ground-truth edge map ($GT_{edge}$) extracted from the labels, which serves as the target for the proposed boundary-aware supervision. The core effectiveness of the module is captured in subfigure (c), which represents the predicted edge intensity (entropy map). In our framework, focal loss is applied to align (c) with (b). Because the edge pixels in (b) are extremely sparse, focal loss ensures that the model is not biased toward overwhelming the background pixels. As shown in (c), the high-entropy regions are precisely localized along the branch boundaries with minimal noise. This high-fidelity edge prediction effectively “guides” the segmentation process in (a), thus preventing the common issue of blurred or fragmented contours in slender structures and ensuring a robust geometric basis for subsequent tasks.

### 2.3. Topology-Guided Structural Filtering

To refine the segmentation mask by removing structurally unstable regions, the system performs a topology analysis on the skeletonized branch mask to identify ambiguous areas such as bifurcations, junctions, and overlapping structures.

**Figure 5:**
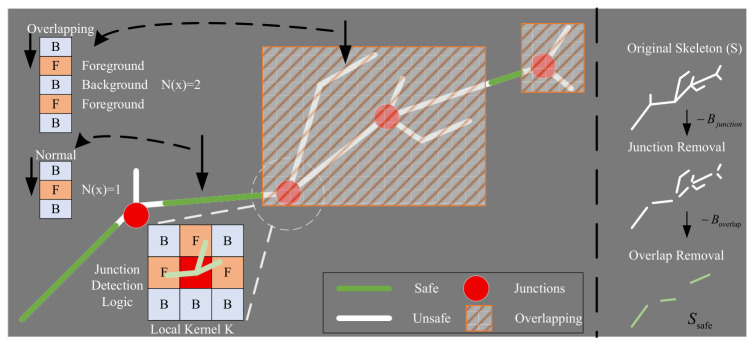
Topology-Aware Structural Analysis.

Let the binary segmentation mask be denoted as M(x,y)∈{0,1}, where M(x,y)=1 indicates the branch pixels. To mitigate small floating noise artifacts generated at longer viewing distances where segmentation boundaries become unstable, a pre-filtering step is applied. A morphological opening filter with a 3×3 elliptical kernel cleans the binary mask, followed by connected component analysis to remove noise artifacts with a contour area smaller than $A_{\min}=150\;\mathrm{pixels}$. A morphological thinning operation (via standard skeletonization) is then applied to the cleaned mask to obtain a one-pixel-wide skeleton representation S=Skeleton(M), which preserves the topological structure of the branch network, as shown in Figure 5.

Branch junctions are detected by applying a local convolution operator to the skeleton map as follows:

(6) R(x,y)=(S∗K)(x,y)

where *K* is a 3×3 neighborhood kernel designed with a central weight of 10 to decoupled central pixel occupancy from surrounding neighbor counts:

(7) $$K=\left[\begin{array}{ccc} 1 & 1 & 1\\ 1 & 10 & 1\\ 1 & 1 & 1 \end{array}\right]$$

Setting the response threshold to R(x,y)≥13 mathematically guarantees that the candidate pixel itself is a valid skeleton point (contributing 10) and has at least three 8-connected neighbors (contributing ≥ 3), which uniquely defines a bifurcating junction or cross-intersection node. These nodes, while topologically significant, are considered structurally unstable for reliable branch representation due to their complex bifurcating geometry and are therefore targeted for removal.

In addition to local junctions, overlapping branches along the viewing direction are detected through column-wise structural analysis. For each image column *x*, the number of foreground transitions is computed as $N\left(x\right)=\sum_{y}I\left(M\left(y+1,x\right)-M\left(y,x\right)>0\right)+M\left(0,x\right)$, where I(·) is the indicator function. A column *x* with N(x)≥2 is classified as containing multi-layer overlapping structures or clutter within a single viewing ray. It should be noted that the column-wise metric N(x) is inherently sensitive to branch orientation and may underestimate horizontal overlaps in a single image. Nevertheless, under the single-view assumption adopted in this work, it provides a simple and computationally efficient criterion for identifying vertically overlapping branch structures.

Guided by the detected overlapping columns and junction regions, to robustly isolate stable branch segments, horizontal removal buffers are constructed. The identified overlapping columns N(x)≥2 are expanded along the horizontal axis using a dilation buffer of radius $r_{x}=30\;\mathrm{pixels}$. Similarly, the detected junction points are dilated using a square kernel of size 30×30pixels ($r_{\mathrm{junc}}=15\;\mathrm{pixels}$). Skeleton pixels falling within these expanded buffers are removed, and tiny residual spur artifacts with an area less than $A_{\mathrm{burr}}=20\;\mathrm{pixels}$ are eliminated from the refined skeleton $S_{\mathrm{filtered}}$.

The final output of this stage is the refined branch mask $M_{\mathrm{filtered}}$ and filtered skeleton $S_{\mathrm{filtered}}$, in which structurally unstable regions—such as bifurcations, junctions, and overlapping areas—have been removed, while topologically reliable branch segments are preserved. This refined mask provides a more reliable structural input for downstream perception tasks. The complete procedure for topology-guided structural filtering is outlined in Algorithm A1.

## 3. Results

This section details the experimental framework designed to validate the proposed methodology. It encompasses dataset preparation, evaluation protocols, and a comprehensive quantitative analysis. The evaluation is structured into two distinct phases: (1) offline assessment of segmentation performance and (2) edge deployment validation. The primary objective is to substantiate the segmentation accuracy and cross-dataset generalization capability of the proposed DDRNet-Branch architecture in complex natural environments characterized by slender branch structures.

### 3.1. Experimental Setup and Evaluation Metrics

#### 3.1.1. Dataset Description

The experimental evaluation utilizes two distinct data sources to ensure a rigorous assessment of the proposed framework.

First, a task-specific dataset is curated to reflect high-precision structural perception requirements in cluttered settings. This collection comprises 757 images sourced from close-range aerial and ground-based captures. The data are characterized by thin-structure objects situated within a 0 to 2 m range from the sensor, providing a challenging benchmark for fine-grained structural identification. The dataset is partitioned into training, validation, and testing sets with a ratio of 7:2:1. Specifically, this results in 530 images for training, 151 images for validation, and 76 images for testing, ensuring a balanced evaluation of the model’s structural perception capabilities.

Second, to benchmark performance against established standards and enhance the generalizability of the findings, the Tree dataset of Urban Street is employed. This public resource contains 1485 high-resolution urban scene images annotated for instance-level segmentation. As detailed in the original dataset [Zhejiang Agriculture and Forestry University, 2022], the images span 13 distinct tree species, captured across diverse seasonal and climatic conditions. The dataset provides 1193 training, 149 validation, and 143 testing images, offering a robust benchmark for pixel-level recognition of elongated structures amidst complex background clutter.

#### 3.1.2. Experimental Setup and Implementation Details

The proposed framework was developed and evaluated on two distinct computing platforms to simulate the transition from offline training to real-time aerial deployment, as summarized in Table 1. Model training was conducted on a high-performance workstation equipped with an Intel Core i9-14900K CPU and an NVIDIA GeForce RTX 4080 GPU, providing the necessary throughput for large-scale architectural optimization. For onboard validation, the model was deployed on an NVIDIA Jetson Orin Nano Edge module. This platform, featuring 1024 CUDA cores and 8 GB of memory, represents a typical resource-constrained environment for UAVs, where inference is executed using FP16 precision to maximize power efficiency and throughput.

To ensure experimental reproducibility and robust convergence, all models were trained from scratch without pretrained weights. We employed the SGD optimizer with a batch size of 4 over 800 epochs. To mitigate the risk of overfitting in complex natural environments, we implemented an EarlyStoppingHook with a patience of 50 epochs and a minimum delta of 0.001 based on validation mIoU. The training pipeline incorporated a rigorous data-augmentation strategy, including random resizing (0.5–2.0 scale), rotations, and photometric distortions (adjustments to brightness, contrast, and hue), which effectively enhanced the model’s invariance to lighting and viewing-angle variations. Furthermore, random seeds were strictly fixed across all experiments to ensure consistency. The software environment utilized PyTorch and CUDA versions customized to respective hardware constraints—specifically CUDA 12.1 for the training workstation and CUDA 11.4 for the edge platform—to ensure optimal kernel execution.

#### 3.1.3. Evaluation Metrics

A robust set of quantitative criteria is indispensable for validating the segmentation efficacy and deployment viability. This study employed four principal metrics: pixel accuracy (Acc), intersection over union (IoU), parameter count (Params), and frames per second (FPS). The fundamental elements for computing these metrics are defined as follows:

True positive (TP): Pixels correctly identified as belonging to the target class.

True negative (TN): Pixels correctly classified as background pixels.

False positive (FP): Background pixels erroneously labeled as the target class.

False negative (FN): Target pixels incorrectly classified as the background.

Pixel accuracy (Acc): quantifies the global ratio of correctly classified pixels relative to the total pixel population. It is mathematically expressed as follows:

(8) $$Acc=\frac{TP+TN}{TP+TN+FP+FN}$$

The derived forms of this metric include the overall accuracy (aAcc) for the entire dataset and the mean accuracy (mAcc), which calculate the average class-specific accuracies to mitigate class-imbalance bias.

Intersection over union (IoU) provides a more stringent measure of spatial overlap between the predicted segmentation mask and the annotated ground truth. For a specific class, it is expressed as

(9) $$IoU=\frac{TP}{TP+FP+FN}$$

The aggregate metric utilized for model comparison was the mean intersection over union mIoU, which was obtained by averaging IoU scores across all semantic categories. A higher mIoU value correlates strongly with superior delineation of branch boundaries and morphology.

Parameters (Params) denotes the sum of all trainable weights and biases within the model architecture. This metric serves as a proxy for the memory footprint and computational storage requirements. For UAV-based perception applications, minimizing the parameter count is critical for enabling efficient inference within the stringent power and memory budgets of embedded flight controllers.

Frames per second (FPS) is the empirical measure of inference throughput and indicates the number of image frames processable by the model per second on the target Jetson AGX Xavier hardware. A high FPS is essential for dynamic visual tracking and real-time structural perception during aerial maneuvers.

### 3.2. Ablation and Comparative Experiments

#### 3.2.1. Ablation Experiment

Ablation experiments were conducted on both the public UrbanStreet Branch dataset [39] and our self-constructed dataset to evaluate the contribution of each proposed component comprehensively. As described in Section 3.1.1, the original UrbanStreet dataset contains multiple tree categories, which we unified into a single-branch class to align with our task setting. This ensures consistency between the public benchmark and our custom dataset, both of which focus on slender-branch segmentation in complex outdoor environments.

Beginning from the DDRNet baseline, we progressively incorporated the proposed modules, including the GCBlock (replacing RB/RBB), SSPPM (replacing DAPPM), and BOM. The quantitative results are summarized in Table 2, which presents the individual and combined contributions of each component. All the models were evaluated with an input resolution of 1024 × 1024. The FPS was measured on a desktop GPU using PyTorch inference, while latency was reported on an NVIDIA Jetson Orin Nano with TensorRT acceleration (FP16, batch size = 1).

Based on Table 2, several clear trends can be observed by comparing representative configurations across the two datasets and hardware platforms. Beginning from the DDRNet baseline, introducing the SSPPM (b) yielded the most significant and consistent improvement in accuracy. On the Drone-Branch dataset, it increased the mIoU from 87.64% to 89.11% (+1.47%), whereas on UrbanStreet, it indicated an improvement by +0.83%. This suggests that the strip-and-serial pooling structure of the SSPPM effectively captures both the close-range, fragmented branch features (0–2 m) in the private dataset and the complex arboreal structures in urban environments. By contrast, the individual contributions of the GCBlock (a) and BOM (c) were relatively limited when used in isolation, thus indicating that multiscale context modeling is the primary factor for improving segmentation e in these challenging scenarios.

When the modules were combined, the interaction between the components revealed the distinct characteristics of the two datasets. For the UrbanStreet dataset, adding the BOM (c) to the baseline or other variants resulted in a significant increase in accuracy (e.g., variant c achieved an mIoU of 89.20%, which represents a +4.2% increase). This is because the UrbanStreet dataset contains high-resolution images with diverse tree species and complex backgrounds, where the boundary-aware supervision of the BOM effectively resolves the semantic ambiguity between branches and urban clutter. On the Drone-Branch dataset, although the single-module gain of the BOM is smaller because of the extremely thin and irregular nature of close-range branches, the combination (bc) achieved the best overall performance (89.96% mIoU, an increase by +2.32% over the baseline). This indicates that the high-level semantics from the SSPPM and the fine-grained edge refinement from the BOM are complementary, thus ensuring both regional consistency and contour sharpness.

In terms of computational efficiency, a significant performance divergence was observed between the PC framework and the embedded Jetson platform. On the desktop GPU using PyTorch, the GCBlock (a) and its combinations (ab, abc) substantially improved the throughput. For instance, the (abc) configuration recorded 221.4 FPS, which is 73.1% higher than that of the baseline. This improvement suggests that the reparameterized design of the GCBlock and the streamlined serial pipeline of the SSPPM effectively simplify the computational graph during PyTorch inference, thus improving memory-access efficiency and reducing kernel overhead. However, on the Jetson Orin Nano, the latency remained relatively stable across all variants (13.7–14.1 ms). This stability is primarily attributed to the deployment-level optimizations of TensorRT. Because TensorRT performs operator fusion and kernel auto-tuning, the structural differences between the training-time multibranch blocks collapse mathematically. Consequently, the final inference speed on the embedded platform becomes more dependent on the hardware peak throughput than on the intermediate graph complexity of the framework.

More importantly, despite these architectural modifications, none of the proposed enhancements introduced additional inference overhead on the embedded platform. All configurations (bc and abc) maintained or even slightly reduced the latency compared with the baseline while achieving higher accuracy. In the most optimized configuration (abc), the model achieved near-peak accuracy on both datasets while remaining within the real-time constraints required for high-frequency aerial perception. These results confirm that the proposed design successfully balances high-precision segmentation for complex structural targets with the stringent efficiency requirements of edge-computing devices, demonstrating its suitability for diverse autonomous aerial monitoring and inspection applications.

#### 3.2.2. Model Comparison Experiment

**Figure 6:**
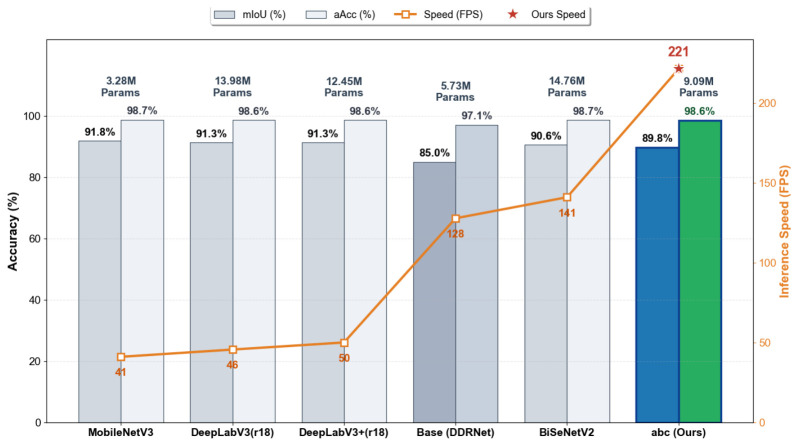
Efficiency comparison of different models in terms of mIoU, FPS, and model size. FPS values are evaluated on a desktop NVIDIA RTX 4080 GPU. The blue bars represent the mIoU of the proposed model, while the green bars represent the aAcc of the proposed model.

To further evaluate the practical advantages of the proposed abc model, we conducted a comparative study against several representative baselines, including heavyweight models DeepLabV3 [40] and DeepLabV3+ [41] (both with a ResNet-18 backbone), the mobile-oriented MobileNetV3 [42] with an L-RASPP head, and the real-time segmentation model BiSeNetV2 [43]. The trade-offs among segmentation accuracy, inference speed, and model complexity are illustrated in Figure 6.

All the models were evaluated under identical experimental settings on the UrbanStreet dataset with a unified input resolution of 1024×1024. Both the mIoU and aAcc have been reported to provide more comprehensive assessments of segmentation performance.

The results revealed clear differences among the compared methods. DeepLabV3 and DeepLabV3+ achieved strong segmentation performance (mIoU above 91% and aAcc of approximately 98.6%), although their inference speeds remained limited to approximately 40–50 FPS on a desktop GPU. This is primarily because of the computational overhead introduced by atrous spatial pyramid pooling and dense feature processing at high resolutions. Similarly, MobileNetV3 achieved competitive accuracy but exhibited comparable latency constraints. Such performance is generally insufficient for high-speed UAV scenarios, where low latency is critical for robust navigation and structural perception tasks.

BiSeNetV2, which is designed for real-time applications, significantly improved the inference speed (over 140 FPS) while maintaining a relatively high accuracy (90.6% mIoU and 98.7% aAcc). However, a clear trade-off remained between speed and fine-grained segmentation quality, particularly for slender branch structures.

By contrast, the proposed abc model achieved an inference speed of 221.41 FPS, thereby outperforming all baselines substantially. This represents a speedup exceeding 4.4× over DeepLabV3+ and a 1.5× improvement over BiSeNetV2 while maintaining competitive accuracy (89.81% mIoU and 98.64% aAcc). Despite exhibiting a slightly lower mIoU (approximately 1–2% compared with the strongest baselines), the model preserved a high overall classification accuracy and significantly enhanced the temporal resolution.

As illustrated in Figure 6, the proposed method achieved a favorable balance between efficiency and accuracy, positioning itself on the optimal tradeoff frontier for autonomous aerial perception. By replacing the standard DAPPM with the serial-structured SSPPM and introducing reparameterized GCBlocks, the model significantly improved the feature representation for slender targets while maintaining superior computational efficiency. This design ensures highly accurate structural segmentation under real-time constraints, leaving sufficient computational capacity for other concurrent onboard perception tasks in edge-computing scenarios.

### 3.3. Qualitative Analysis of Topology-Guided Structural Filtering

**Figure 7:**
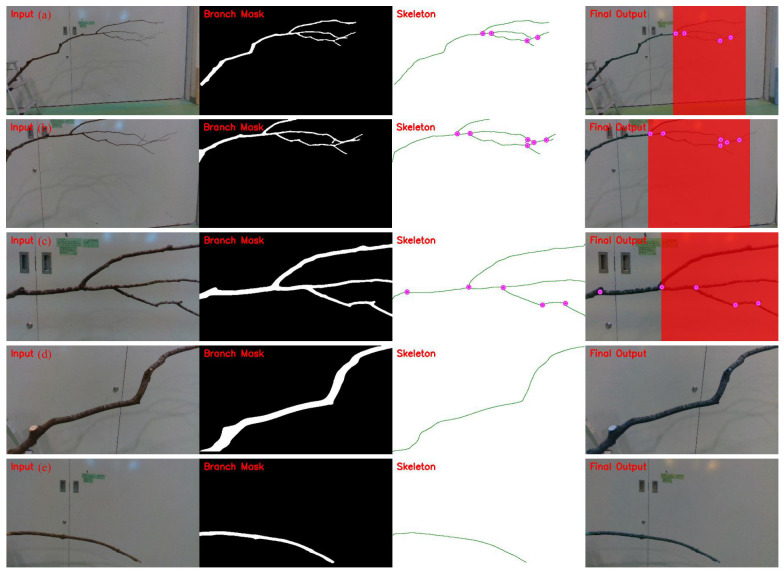
Qualitative results on controlled indoor scenes. Columns from left to right: input RGB image, binary segmentation mask, skeleton representation, and final filtered output. In the final output, red shaded regions indicate multibranch areas removed by topology analysis, and pink circles denote detected junction nodes. Rows (**a**–**e**) correspond to: (**a**) global view; (**b**) close-up view of a bifurcation structure; (**c**) another bifurcation example showing junction detection; (**d**) curved trunk without junctions; (**e**) horizontal straight branch without junctions.

**Figure 8:**
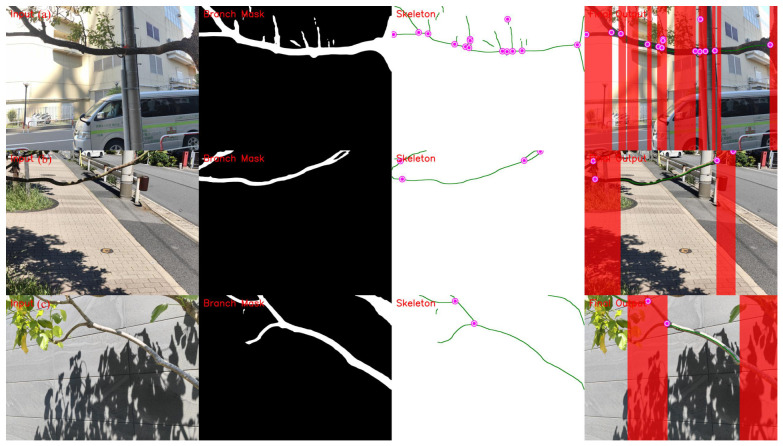
Qualitative results on unconstrained outdoor scenes. Columns from left to right: input RGB image, binary segmentation mask, skeleton representation, and final filtered output. The shaded red regions indicate areas removed by topology analysis, and pink circles denote detected junction nodes. Rows (**a**–**c**) correspond to natural tree branches under varying lighting conditions, leaf occlusions, and complex background foliage.

To validate the effectiveness of the proposed topology-guided structural filtering framework, we present qualitative results under diverse structural configurations, observation distances, and environmental conditions. The evaluation is organized into two complementary groups: controlled indoor scenes (Figure 7) for systematic assessment of geometric reasoning, and unconstrained outdoor scenes (Figure 8) for generalization validation.

The framework maintains consistent topology-aware filtering across different observation distances. In the indoor global view (Figure 7a), the segmentation mask exhibits minor fragmentation on distant, slender branches due to limited pixel resolution—an inherent limitation of fixed-resolution sensing. The close-up view (Figure 7b) shows the same bifurcation structure at a closer distance, confirming that the method captures intrinsic topological properties rather than relying on pixel-level heuristics. In both cases, the junction regions are reliably identified and marked for removal, demonstrating consistent behavior across scale variations.

Figure 7c presents a bifurcation structure where multiple branches intersect. The skeleton representation clearly reveals the junction node (marked in pink), and the corresponding region in the original mask is removed (shaded in red). It should be noted that the junction node exhibits a slight positional offset when mapped back to the original image coordinates. This offset originates from the inherent approximation of the morphological skeletonization process, which may introduce minor localization errors at complex topological transitions. To compensate for this, the removal buffers are deliberately expanded during the filtering stage, ensuring that all structurally ambiguous areas are conservatively excluded even in the presence of such offsets.

As shown in Figure 7d,e, the framework correctly identifies curved and horizontal branch segments that contain no junctions or overlapping structures. In both cases, the skeleton representation remains continuous and free of bifurcation nodes, and the final filtered mask preserves the complete branch structure without any removal. This confirms that the method does not over-filter geometrically stable regions, distinguishing it from naive morphological operations that might indiscriminately erode branch endpoints.

Unlike the clean binary masks obtained in indoor settings, the outdoor segmentation results (Figure 8a–c) exhibit significantly more fragmentation. This degradation stems from multiple factors: complex lighting conditions (strong sunlight and deep shadows), dense leaf occlusions, and background foliage with similar color and texture to target branches. Despite these challenges, the proposed framework consistently identifies and removes junction and overlapping regions. The skeletonization process abstracts the fragmented mask into a structural representation, and the topology analysis detects branch junctions and multi-branch areas based on local connectivity. This filtering mechanism operates directly on the structural relationships within the mask—regardless of segmentation imperfections—and removes the corresponding ambiguous areas. Consequently, even when occlusion causes the segmentation mask to partially lose the structural integrity of junction or multi-branch regions, the topology analysis still identifies the remaining structural ambiguities and excludes them from the final output. This ensures that a substantial portion of potentially hazardous areas are filtered out, thereby enhancing safety. While this conservative strategy may occasionally remove structurally stable regions that happen to exhibit junction-like connectivity, it substantially reduces the risk of retaining hazardous multi-branch areas in the final output, thereby improving the overall reliability of the refined mask for downstream applications.

It is also worth noting that in the final filtered outputs shown in both indoor and outdoor scenes (Figure 7 and Figure 8), the preserved stable regions consistently exhibit sufficient clearance above and below the branch structures in the unmasked areas. This observation suggests that the refined masks not only remove structurally ambiguous regions but also retain geometrically reliable branch segments with adequate surrounding free space, which is a desirable property for generating geometrically consistent candidate regions that may benefit downstream navigation, planning, or interaction modules.

Overall, the qualitative results confirm that the proposed topology-guided structural filtering framework provides robust branch mask refinement across both controlled and unconstrained conditions, consistently removing structurally ambiguous regions while preserving topologically reliable branch segments.

**Figure 9:**
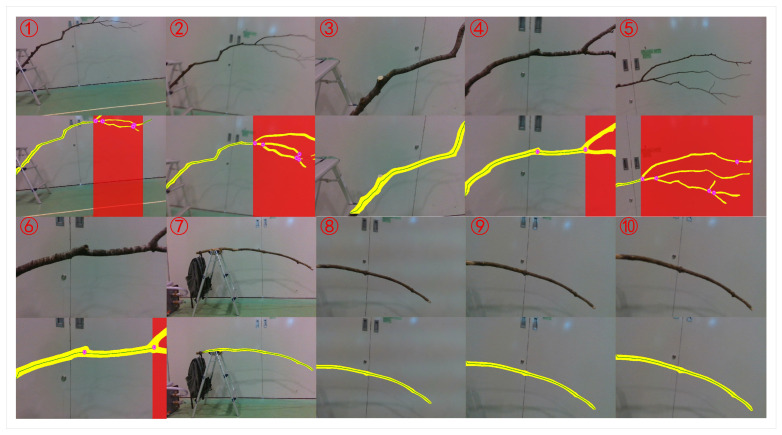
Representative quantitative validation examples corresponding to the measurements reported in Table 3. The first and second rows show the original images and the corresponding topology-guided filtering results for Images 1–5, respectively. The third and fourth rows present the original images and filtering results for Images 6–10. Yellow regions indicate the segmented branch masks, green lines represent the extracted branch skeletons, pink circles denote detected junction nodes, and red shaded regions indicate removed overlapping branch areas.

To further complement the qualitative observations, a quantitative evaluation of the proposed topology-guided structural filtering framework was conducted on 10 representative branch images captured at observation distances ranging from 0.5 m to 2.5 m. For each image, the branch centerline was manually annotated to obtain the ground-truth skeleton length. The extracted skeleton length before filtering and the remaining safe branch length after topology-guided filtering were then measured automatically. In addition, branch junctions compared with the detected junctions, while the removed overlap area was computed from the filtered regions. Representative examples are shown in Figure 9, and the corresponding quantitative results are summarized in Table 3.

As summarized in Table 3, the proposed topology-guided structural filtering framework exhibits consistent behavior across representative branch configurations captured at different observation distances. In images containing no or only negligible overlapping regions, the removed overlap area remains close to zero and the safe branch ratio consistently exceeds 98%, indicating that the proposed filtering introduces minimal influence on geometrically stable branch segments while preserving their structural continuity.

In contrast, scenes containing dense branch intersections exhibit substantially larger removed overlap areas and correspondingly lower safe branch ratios. This behavior is expected because the proposed framework intentionally removes structurally ambiguous multi-branch regions rather than preserving the complete branch skeleton. Consequently, only geometrically reliable branch segments are retained for subsequent candidate region generation, while potentially hazardous overlapping regions are conservatively excluded.

The junction statistics further demonstrate that the proposed topology analysis correctly identifies most branch junctions under different observation distances and structural complexities. The few false detections mainly occur when branches located at different depths appear to overlap in the monocular image due to perspective projection. Although these branches are physically separated, their projected skeletons become locally connected, leading to occasional false junction detections. Similarly, a small number of missed junctions occur in highly cluttered regions where severe occlusion partially interrupts the skeleton connectivity. Since the subsequent filtering stage adopts a conservative overlap-removal strategy, these occasional errors have only a limited influence on the final filtered branch regions.

Overall, the quantitative evaluation is consistent with the qualitative observations presented in Figure 7, Figure 8 and Figure 9. Together, these results provide additional evidence that the proposed topology-guided structural filtering framework effectively identifies branch junctions and overlapping regions while preserving stable branch structures suitable for downstream candidate region generation.

### 3.4. Edge Deployment Performance

To further evaluate the practicality of the proposed method for onboard UAV perception, deployment experiments were conducted on an NVIDIA Jetson Orin Nano using TensorRT with FP16 optimization. All models were evaluated using the original input resolution of 1280 × 720 without image downsampling to reflect realistic UAV perception scenarios, where preserving fine branch structures is critical. Besides segmentation accuracy, inference speed, latency, GPU memory consumption, and TensorRT engine size were measured to assess deployment efficiency under embedded computing constraints.

Table 4 summarizes the deployment performance of different segmentation networks. Compared with the baseline model, the proposed abc_branch improves the segmentation accuracy from 86.06% to 87.68%, while simultaneously increasing the inference speed from 96.17 FPS to 99.43 FPS. The inference latency is reduced from 10.40 ms to 10.06 ms. Moreover, the peak GPU memory decreases from 78.93 MB to 64.66 MB, corresponding to an 18.1% reduction, while the average runtime memory decreases from 402 MB to 332 MB (17.4% reduction). The TensorRT engine size is also reduced to a compact size, making the model well-suited for edge deployment.

The advantage of the proposed method becomes more evident under high-resolution inference. Although DeepLabV3 and DeepLabV3+ achieve slightly higher mIoU values, their inference speeds decrease to only 18.73 FPS and 14.46 FPS, respectively, with inference latencies exceeding 50 ms, making them less suitable for real-time onboard perception. BiSeNet achieves competitive segmentation accuracy but operates at only 35.71 FPS while requiring substantially higher runtime memory. MobileNetV3 exhibits the lowest peak memory consumption; however, its inference speed drops to only 7.88 FPS. This behavior is mainly attributed to its extensive use of depthwise separable convolutions and channel attention modules, whose hardware utilization becomes less efficient for high-resolution semantic segmentation on embedded GPUs. Consequently, despite its lightweight architecture, MobileNetV3 does not provide a speed advantage under the adopted deployment setting.

Overall, the proposed abc_branch achieves the highest inference throughput while maintaining competitive segmentation accuracy and reduced memory consumption. These results demonstrate that the proposed method provides an effective balance between perception accuracy and computational efficiency for real-time UAV onboard branch perception.

### 3.5. Post-Processing Efficiency Analysis

To evaluate the computational efficiency of the proposed topology-guided post-processing pipeline, we conducted timing tests at 720 × 1280 resolution using simulated branch masks with varying densities. Density is defined as the percentage of branch pixels in the image, ranging from 10% to 80% to cover typical operational scenarios. The test masks were generated to simulate realistic branch configurations, where low density (10%) corresponds to distant slender branches with more bifurcations (10 branches), while high density (80%) corresponds to close thick branches with fewer branches (2 branches). Each density level was tested 30 times, and the average processing time was reported.

As shown in Table 5, the post-processing pipeline consists of five core steps: mask preprocessing (morphological opening and small component filtering), skeletonization (Zhang-Suen thinning algorithm), junction detection (branch point detection using convolution kernel), overlap detection (column-wise transition analysis), and junction removal (removal of junction and overlap regions).

Experimental results reveal that skeletonization is the computational bottleneck, accounting for over 89% of total processing time across all densities, reaching up to 98.4%. At 10% density, skeletonization takes 224.06 ms, accounting for 89.7% of total time. When density increases to 30%, skeletonization time surges to 1400.91 ms, accounting for 98.4%. This substantial increase occurs because the Zhang-Suen thinning algorithm requires iterative scanning of all foreground pixels until convergence, with the number of iterations increasing with connected component size and structural complexity. At 30% density, the mask contains both a large number of pixels (277,889) and multiple branches (6), creating a computational peak. Interestingly, at higher densities (50% and 80%), although individual branches become thicker, the reduced number of branches (4 and 2, respectively) leads to fewer connected components, resulting in lower skeletonization times (1122.73 ms and 493.53 ms) compared to the 30% density case.

The preprocessing step exhibits stable performance, taking approximately 5–8 ms across all densities, accounting for less than 1% of total time. This step includes morphological opening and connected component filtering, whose computational complexity is proportional to the number of foreground pixels but does not involve iterative operations. The remaining three steps—junction detection, overlap detection, and junction removal—together take approximately 12–17 ms, accounting for about 1–2% of total time. These steps operate on the skeletonized output and involve only single-pass convolution, transition counting, and dilation operations, making them computationally lightweight.

Overall, the timing analysis identifies skeletonization as the dominant component in the post-processing pipeline, accounting for the majority of processing time. Nevertheless, the overall framework remains practical for real-world deployment—the remaining topology-guided filtering steps add negligible overhead (less than 18 ms), and the total post-processing latency is comparable to the segmentation front-end. This decomposition provides a clear direction for future optimization without undermining the practical viability of the current approach.

## 4. Discussion

This paper presented a topology-guided branch perception framework for UAV perception in natural environments, combining an improved real-time semantic segmentation network with topology-guided structural filtering. To enhance segmentation performance on slender branch structures, we introduced three complementary modules: GCBlock for efficient feature extraction, SSPPM for anisotropic multiscale context modeling, and BOM for boundary refinement. Experimental results on both the Drone-Branch and UrbanStreet datasets demonstrated that the proposed framework achieved consistent improvements in segmentation accuracy. The proposed segmentation network maintained real-time inference performance on embedded hardware, while the subsequent topology-guided filtering improved the structural reliability of the segmentation results. In particular, SSPPM provided the largest contribution to segmentation accuracy, whereas BOM further improved boundary quality and structural consistency without increasing deployment latency.

Building upon the segmentation results, the proposed topology-guided post-processing pipeline refines pixel-level masks by exploiting branch topology and structural connectivity. This process effectively suppresses structurally unstable regions—such as bifurcations, junctions, and overlapping structures—from the segmentation output, yielding refined masks with improved structural reliability for downstream perception tasks.

From a perception perspective, the preserved stable regions in the filtered masks are particularly noteworthy. As observed in the qualitative results (Figure 7 and Figure 8), the retained branch segments generally exhibit sufficient surrounding free space above and below the branches in the image plane. This observation suggests that the proposed topology-guided filtering removes structurally ambiguous regions while preserving geometrically consistent branch segments. Such refined masks may provide more reliable candidate regions for downstream modules, including motion planning, navigation, or physical interaction. However, the effectiveness of these downstream applications requires dedicated validation and is beyond the scope of this work.

Despite the promising results, the proposed framework has several limitations that should be acknowledged. First, the topology analysis relies entirely on the quality of the input segmentation mask. In cases where the segmentation network fails to detect branches due to severe occlusion, extreme lighting conditions, or very low contrast, the subsequent skeletonization and topology analysis cannot recover the missing structures. While the framework demonstrates robustness to moderate segmentation degradation, its performance inevitably degrades when the input mask contains substantial omissions. Second, the conservative filtering strategy, while effective at reducing the risk of retaining hazardous multi-branch areas, may occasionally remove structurally stable regions that exhibit junction-like connectivity. This trade-off between recall and precision is intentional—prioritizing safety over completeness—but may be suboptimal for applications requiring full structural reconstruction. Third, the current framework operates on a per-frame basis and does not incorporate temporal information to leverage consistency across consecutive frames. As a result, the filtering decisions are made independently for each frame, which may lead to minor inconsistencies when applied to video sequences with rapid motion or frequent occlusions.

Based on these limitations, we identify the following near-term research directions for direct improvement. First, we plan to incorporate temporal information into the filtering pipeline by leveraging inter-frame consistency. This would enable the framework to maintain stable filtering decisions across consecutive frames and potentially recover temporarily occluded structures through temporal aggregation. Second, we intend to extend the current framework to operate on 3D point cloud representations derived from depth sensing. This would allow the use of geometric information—such as branch thickness, spatial separation, and three-dimensional connectivity—to complement the 2D topology analysis, particularly in scenarios with severe occlusion or ambiguous depth projection. Third, we aim to explore adaptive thresholding strategies that adjust the removal buffer size based on local structural characteristics, such as branch density or local curvature, rather than using a fixed dilation radius. This would reduce the likelihood of over-filtering stable regions while maintaining the conservative removal of genuinely ambiguous areas. Fourth, we plan to extend the current single-frame framework to a multi-view perception system by integrating active viewpoint adjustment and temporal multi-view association on an actual UAV platform. Such an extension would enable the UAV to observe target branches from complementary viewpoints through coordinated flight and gimbal motion, thereby alleviating the orientation sensitivity of the current column-wise overlap analysis. We also plan to investigate viewpoint-selection strategies and validate the proposed approach through real UAV flight experiments in forest environments under varying illumination, ego-motion, and occlusion conditions.

Regarding computational efficiency, our timing analysis identifies skeleton extraction as the primary bottleneck in the current topology-guided post-processing pipeline, accounting for over 89% of the total post-processing time and preventing the complete end-to-end framework from operating in real time. We plan to investigate GPU-accelerated parallel implementations of thinning algorithms, or alternative approaches such as Guo-Hall, Hilditch, K3M, distance-transform-based, or potential-field-based methods to substantially reduce the processing latency for embedded deployment.

Beyond the above limitation-driven improvements, the framework can be further extended along several complementary directions to address broader operational scenarios and system-level challenges.

Regarding multi-UAV collaborative perception, the proposed single-agent pipeline could be extended to a distributed setting where multiple aerial platforms share structural environmental priors to compensate for individual occlusions and expand perceptual coverage. A federated learning-based distributed control paradigm [44] could enable collaborative model refinement without aggregating raw sensory data across agents, thereby preserving communication bandwidth and data privacy in large-scale forest monitoring missions.

Regarding perception robustness under aggressive maneuvers, the current RGB-only module is susceptible to motion blur and rapid illumination changes. We plan to integrate inertial and star-sensor data to provide reliable attitude priors, following advanced inertial/star sensor fusion methods [45] that have demonstrated effectiveness under dynamic conditions. Such attitude-aware information could be fed into a spatial transformer network to rectify distorted features before segmentation, thereby improving mask consistency during rapid rotations or high-speed flights.

Regarding system-level reliability, we will develop a real-time perception health-monitoring module that evaluates the temporal consistency and area continuity of the filtered masks. Drawing upon fault diagnosis principles for redundant systems [46], this module could detect abrupt degradation patterns and trigger fail-safe responses—such as hovering, ascending, or reinitializing the perception pipeline—to ensure robust operation in safety-critical scenarios.

Regarding the generalization of structural reasoning, while the current topology analysis relies on handcrafted skeleton features, these heuristic rules may not optimally generalize to highly diverse or extremely dense foliage. We will explore a learning-based alternative, such as a category-guided graph convolution network [47], which constructs graph representations from skeleton endpoints and junction candidates to learn stable versus unstable region classification directly from data. This data-driven paradigm has the potential to reduce false-positive removals and improve junction detection accuracy under severe clutter.

Finally, we note that while the current study focuses on perception and structural filtering, the proposed framework could also serve as a structural prior for broader robotic applications that require reliable branch-level environmental understanding. For instance, in tasks such as aerial grasping or perching on branches, the refined mask and topology information provided by our method could inform target selection and contact planning. Similarly, in forest monitoring and inspection scenarios, the framework could support fine-grained structural mapping of vegetation. Exploring such integrations remains an interesting direction for future research.

## Figures and Tables

**Table 1:** Summary of development and deployment environments.

Model Training Workstation			
CPU	Intel Core i9-14900K	Optimizer	SGD
GPU	NVIDIA RTX 4080	Batch Size	4
CUDA	12.1	PyTorch	2.5.1
Python	3.10	Epochs	800
Edge Inference Platform (UAV Onboard)			
Device	Jetson Orin Nano	CUDA	11.4
JetPack	5.1.3	PyTorch	2.1
Python	3.8	Precision	FP16

**Table 2:** Ablation-study results on Drone-Branch and UrbanStreet datasets.

Model			Efficiency			Drone-Branch			UrbanStreet		
a	b	c	Params(T/D)/M	${\mathrm{FPS}}_{\mathrm{PC}}$	${\mathrm{latency}}_{\mathrm{Jet}}$/ms	mIoU	mAcc	aAcc	mIoU	mAcc	aAcc
			5.73/5.73	127.9	14.135	87.64	94.57	99.00	85.00	91.70	97.12
✓			20.69/9.21	149.6	13.734	87.58	94.74	98.97	84.91	91.61	96.83
	✓		5.61/5.61	186.9	13.877	89.11	95.53	99.13	85.83	92.38	97.29
		✓	5.73/5.73	126.4	14.15	87.91	95.44	98.99	89.20	95.74	97.95
✓	✓		20.57/9.09	214.4	13.752	88.43	95.49	99.05	84.95	92.07	96.94
	✓	✓	5.61/5.61	188.3	13.953	89.96	95.73	99.12	89.89	95.96	98.11
✓	✓	✓	20.57/9.09	221.4	13.769	89.94	95.62	99.05	89.81	95.91	98.09

a: GCBlock, b: SSPPM, c: BOM. A check mark (✓) indicates that the corresponding module is included in the model configuration for that experiment. Params (T/D): Training and deployment parameters (M).

**Table 3:** Quantitative validation of topology-guided structural filtering.

Image	Distance (m)	GT (px)	Extracted (px)	Safe Ratio (%)	Junctions	Removed Overlap (px^2^)
1	2.5	1625	1368	49.2	4/4/1/1	244,641
2	2.0	2554	2195	34.7	4/5/1/0	356,131
3	1.5	3202	3014	7.2	0/0/0/0	648,415
4	1.0	1690	1655	63.5	1/2/1/0	165,889
5	0.8	1472	1459	79.0	5/5/0/0	74,031
6	0.5	1245	1179	98.3	1/2/1/0	0
7	2.0	1009	941	99.3	0/0/0/0	59
8	1.5	983	936	99.4	0/0/0/0	0
9	1.0	1157	1095	100.0	0/0/0/0	0
10	0.8	1242	1165	99.7	0/0/0/0	0

Notes: Skeleton lengths are measured in image pixels. The safe branch ratio is computed with respect to the extracted skeleton before topology-guided filtering. Junction statistics are obtained by manual annotation and reported as GT/Detected/False/Missed, where GT denotes the manually annotated junction number, Detected denotes the detected junction number, False denotes falsely detected junctions, and Missed denotes manually annotated junctions that were not detected.

**Table 4:** Deployment performance comparison on the NVIDIA Jetson Orin Nano.

Model	mIoU (%)	FPS	Latency (ms)	Peak Memory (MB)	Runtime Memory (MB)	Engine Size (MB)
Base_branch	86.06	96.17	10.40	78.93	402	11.75
abc_branch (Ours)	87.68	99.43	10.06	64.66	332	17.87
BiSeNet_branch	87.75	35.71	28.00	100.01	496	7.55
DeepLabV3+	87.83	14.46	69.14	91.88	572	23.85
DeepLabV3	87.70	18.73	53.40	96.57	526	26.76
MobileNetV3	87.71	7.88	126.85	57.00	730	7.85

Note: All models were evaluated using TensorRT FP16 optimization with an input resolution of 1280 × 720.

**Table 5:** Post-processing time breakdown at different branch densities.

Density	Branches	Pixels	Processing Time (ms)				
			Preprocess	Skeletonize	Junction	Overlap	Removal
10%	10	95,663	8.22	224.06	5.56	4.64	7.33
20%	8	188,024	6.94	753.67	4.80	3.85	7.47
30%	6	277,889	6.24	1400.91	5.01	4.08	7.48
50%	4	203,619	5.47	1122.73	4.66	3.83	6.70
80%	2	92,401	5.25	493.53	4.70	3.95	5.59

## Data Availability

The original contributions presented in this study are included in the article. Further inquiries can be directed to the corresponding author.

## Appendix A

**Algorithm A1** Topology-Guided Structural Filtering
Require: Raw binary mask *M*, Pre-filtering min area $A_{\min}=150\;\mathrm{px}$, Junction threshold $R_{\mathrm{thresh}}=13$, Buffer radius $r_{x}=30\;\mathrm{px}$, Minimum spur area $A_{\mathrm{burr}}=20\;\mathrm{px}$ Ensure: Refined skeleton map $S_{\mathrm{refined}}$ 1: **Pre-processing:** $M_{\mathrm{clean}}\leftarrow \mathrm{MorphologicalOpen}\left(M,\mathrm{kernel}=3\times 3\right)$ 2: $M_{\mathrm{clean}}\leftarrow \mathrm{RemoveConnectedComponents}\left(M_{\mathrm{clean}},\mathrm{area}\leq A_{\min}\right)$ 3: $S\leftarrow \mathrm{Skeletonize}(M_{\mathrm{clean}})$ ▹ Obtain 1-pixel wide skeleton via thinning 4: **Junction Detection:** 5: $K\leftarrow \left[\begin{array}{ccc} 1 & 1 & 1\\ 1 & 10 & 1\\ 1 & 1 & 1 \end{array}\right]$ 6: R←(S∗K) ▹ Apply 2D spatial convolution 7: $J\leftarrow (R\geq R_{\mathrm{thresh}})$ ▹ Identify bifurcation nodes with ≥3 neighbors 8: **Overlap Detection:** 9: **for** each column *x* in $M_{\mathrm{clean}}$ **do** 10: $N\left(x\right)\leftarrow \sum_{y}I\left(M_{\mathrm{clean}}\left(y+1,x\right)-M_{\mathrm{clean}}\left(y,x\right)>0\right)+M_{\mathrm{clean}}\left(0,x\right)$ 11: **end for** 12: $B_{\mathrm{overlap}}\leftarrow \mathrm{DilateHorizontal}\left(N\left(x\right)\geq 2,\mathrm{radius}=r_{x}\right)$ 13: **Buffer Mask Creation & Filtering:** 14: $B_{\mathrm{junc}}\leftarrow \mathrm{DilateSquare}\left(J,\mathrm{size}=r_{x}\times r_{x}\right)$ 15: $S_{\mathrm{safe}}\leftarrow S$ 16: $S_{\mathrm{safe}}\left[:,B_{\mathrm{overlap}}==1\right]\leftarrow 0$ ▹ Clear skeleton in overlapping columns 17: $S_{\mathrm{safe}}\left[B_{\mathrm{junc}}==1\right]\leftarrow 0$ ▹ Clear skeleton near junctions 18: **Post-processing:** 19: $S_{\mathrm{refined}}\leftarrow \mathrm{RemoveConnectedComponents}\left(S_{\mathrm{safe}},\mathrm{area}\leq A_{\mathrm{burr}}\right)$ 20: **return** $S_{\mathrm{refined}}$
